# Could petroleum biodegradation be a joint achievement of aerobic and anaerobic microrganisms in deep sea reservoirs?

**DOI:** 10.1186/2191-0855-1-47

**Published:** 2011-12-23

**Authors:** Georgiana F da Cruz, Suzan P de Vasconcellos, Célio FF Angolini, Bruna M Dellagnezze, Isabel NS Garcia, Valéria M de Oliveira, Eugenio V dos Santos Neto, Anita J Marsaioli

**Affiliations:** 1 Chemistry Institute, University of Campinas - UNICAMP, POB 6154, 13084-971 Campinas, SP, Brazil; 2 Research Center for Chemistry, Biology and Agriculture (CPQBA), University of Campinas - UNICAMP, POB 6171, 13081-970 Campinas, SP, Brazil; 3 Petrobras R&D Center, Cidade Universitária, Quadra 7, 21949-900 Rio de Janeiro, RJ, Brazil; 4 Engineering and Oil Exploration Laboratory, State University of North Fluminense, 27925-310 Macaé, RJ, Brazil

**Keywords:** Petroleum biodegradation, oxic environment, anoxic environment, 16S rRNA gene, petroleum biomarkers.

## Abstract

Several studies suggest that petroleum biodegradation can be achieved by either aerobic or anaerobic microorganisms, depending on oxygen input or other electron acceptors and appropriate nutrients. Evidence from in vitro experiments with samples of petroleum formation water and oils from Pampo Field indicate that petroleum biodegradation is more likely to be a joint achievement of both aerobic and anaerobic bacterial consortium, refining our previous observations of aerobic degradation. The aerobic consortium depleted, in decreasing order, hydrocarbons > hopanes > steranes > tricyclic terpanes while the anaerobic consortium depleted hydrocarbons > steranes > hopanes > tricyclic terpanes. The oxygen content of the mixed consortia was measured from time to time revealing alternating periods of microaerobicity (O_2 _~0.8 mg.L^-1^) and of aerobicity (O_2_~6.0 mg.L^-1^). In this experiment, the petroleum biodegradation changed from time to time, alternating periods of biodegradation similar to the aerobic process and periods of biodegradation similar to the anaerobic process. The consortia showed preferences for metabolizing hydrocarbons > hopanes > steranes > tricyclic terpanes during a 90-day period, after which this trend changed and steranes were more biodegraded than hopanes. The analysis of aerobic oil degrading microbiota by the 16S rRNA gene clone library detected the presence of *Bacillus*, *Brevibacterium*, *Mesorhizobium *and *Achromobacter*, and the analysis of the anaerobic oil degrading microbiota using the same technique detected the presence of *Bacillus *and *Acinetobacter *(facultative strains). In the mixed consortia *Stenotrophomonas*, *Brevibacterium*, *Bacillus*, *Rhizobium*, *Achromobacter *and 5% uncultured bacteria were detected. This is certainly a new contribution to the study of reservoir biodegradation processes, combining two of the more important accepted hypotheses.

## Introduction

Much of the petroleum of the Campos Basin has been altered by microbial degradation decreasing the value of the residual oil. This is a major problem for the global economy (Magot et al. 2000; Cord-Ruwich et al. 1987; Connan 1984; Head et al. 2003). The action of aerobic bacteria (Roling et al. 2003; Orphan et al. 2000; Borzenkov et al. 2006) in petroleum reservoirs is supported by the presence of sufficient oxygen and of nutrient supplies provided by meteoric waters (Connan 1984; Voordouw et al. 1996). The rapid biodegradation of *n*-alkanes and other biomarkers has been reported with pure aerobic cultures as well as with consortia (da Cruz et al. 2008).

However, biodegradation of crude oils has also been observed in the absence of oxygen and anaerobic heterotrophic microorganisms requiring nitrate, sulfate, iron, manganese or carbon dioxide as electron acceptors (Borzenkov et al. 2006; Palmer 1993; Taylor et al. 2001) are responsible for crude oil biodegradation (Rahman et al. 2002; Balk 2007, Jones et al. 2008; Wilkes et al. 2000). Addtionally, Jones et al. (2008) observed sequential removal of hydrocarbons under methanogenic conditions after 686 days.

**Figure 1:**
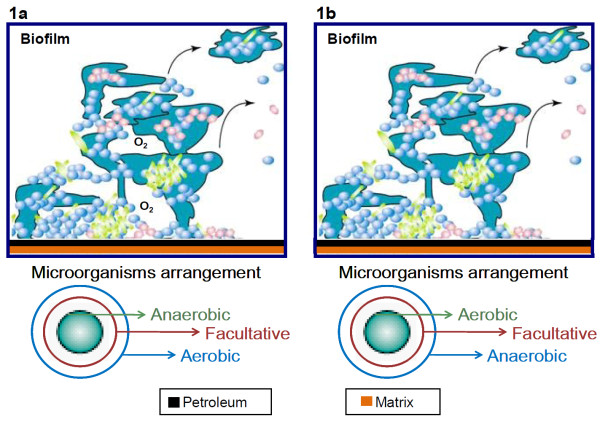
**Proposed arrangement of microorganisms in biofilms in an oxic environment (a) and in an anoxic environment (b)**.

Most studies utilize either aerobic or anaerobic microbial consortia separately, however the existence of mixed microbiota in oil reservoirs with both aerobic and anaerobic consortia actively participating of the biodegradation process should be considered. In subsurface conditions anaerobic microorganisms could provide microquantities of oxygen to the aerobic microbiota by reducing salts like sulfate, nitrate or perchlorate, e.g. ClO_4_^- ^→ ClO_3_^- ^→ ClO_2_^- ^→ Cl^- ^+ O_2 _(Coates 1999; Derek 2000; Xu et al. 2004). This oxygen might be trapped into biofilms and build micro aerobic environments, stimulating aerobic bacterial growth and an aerobic biodegradation lifecycle under a specific microbial arrangement (Shen 1996, da Cruz 2010, Figure 1a). Gradual consumption of the oxygen content would generate an anoxic atmosphere and the beginning of an anaerobic lifecycle following a different microbial arrangement (Figure 1b). The aerobic and anaerobic microorganisms might contribute to a stepwise biodegradation in such a way as to survive in the predominant environment (aerobic and anaerobic).

The goal of the present study was to investigate the potential of aerobic bacteria, anaerobic bacteria and mixed consortia obtained from samples of petroleum formation water and oils from Pampo Field (Brazil) to degrade petroleum biomarkers.

## Methods

### Geological settings

**Figure 2:**
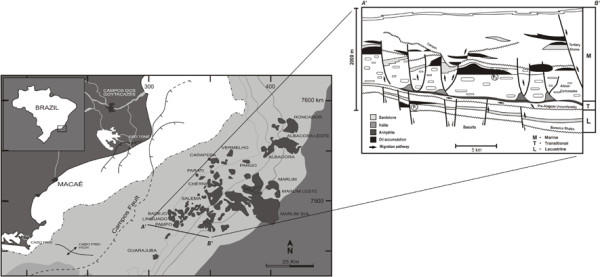
**Location of Campos Basin and oil fields**. Isolines represent water depth of ocean floor (left). Schematic geological section showing model of oil migration and accumulation in the Campos Basin (right). Marine, transitional and lacustrine refer to the major sedimentary sequences based on the respective depositional environments. P1 and P2 refer to samples described in the text.

Pampo Sul is one of the giant offshore oil fields in Campos Basin, one of the most prolific oil basins in Brazil, holding about 85% of total Brazilian oil reserves (before the pre-salt reservoir discovery) and covering an area of about 100,000 km^2^, mostly offshore to the 3400 m isobath (Jahnert et al. 1998). The origin of the Campos Basin is related to the Early Cretaceous break up of the Gondwanaland supercontinent (Rangel et al. 1994). The hydrocarbon source potential of these source rocks is very high, as indicated by carbon contents that average 2-6%, but having intervals reaching values as high as 9-12% (Guardado et al. 2000). The lacustrine saline sediments of the Lagoa Feia Formation (source rock) were interpreted as the source for the typical "Campos oils", including the samples P1 (Coquinas reservoir in Lagoa Feia Formation, classified as not biodegraded) and P2 (Calcarenites reservoir in Macaé formation, classified as biodegraded) (Mello et al. 1988; Mohriak et al. 1989), see Figure 2. Among the diagnostic features for these oil types are a high hopane/sterane ratio (H/S > 5), and predominance of C_27 _steranes. In non-biodegraded oil P1, the bimodal distribution of *n*-alkanes is conspicuous with predominance of the low molar mass fraction (>*n*C_15-_) and a pristane/phytane ratio > 1. More details are given in Table 1.

**Table 1:** Geological and geochemical bulk data.

Oil	Depth interval (m)	Age	Temp. (°C)	Reservoir	S (%)	Sat. HCs (%)	Arom. HCs (%)	NSOs (%)	° API	δ^1^C_VPDB (‰)_
P1	2812-2821	Early Cretaceus	85	Lagoa Feia Formation (coquinas)	0.18	50.70	25.35	23.94	30.7	-23.61
P2	2088-2100	Early Cretaceus	71	Macaé Formation (calcarenites)	0.65	42.55	23.68	33.77	19.7	-24.52

### Microcosms

The aerobic experiment was carried out following the methodology previously described by da Cruz et al. (2008). The aerobic biodegradation process was stimulated by adsorbing oil on sterilized sand and by using 2 g wet cells/30 mg P1 oil/40 mL aqueous Zinder medium (Zinder et al. 1984). The cells were centrifuged from the enrichment experiment as previously described (da Cruz et al. 2008). Aerobic consortia was set up in Erlenmeyer flasks incubated in a shaker with temperature and agitation control (30°C and 150 rpm) during 60 days. Laboratory anaerobic and mixed (aerobic/anaerobic) consortium experiments were set up in 50 mL glass serum bottles sealed with butyl stoppers and aluminium crimps containins resazurin as redox potential control. The bottles were incubated at 30°C during 30 days (with agitation control) and then at 55°C for up to 180 days (without stiring). A gas system fitted with an oxygen sensor and with a regulated atmosphere of nitrogen (80%) and carbon dioxide (20%) was used in the preparation and incubation of the anaerobic and mixed microorganism consortium. Each consortium biodegradation experiment was composed of 40 mL of Zinder nutrient medium (Zinder et al. 1984) containing sources of organic substrates, nitrogen, phophorus, vitamins and trace minerals, made up in Milli-Q water and 30 mg of non-biodegraded oil (P1 from Pampo Sul Field, Campos Basin, Brazil). For the control experiments the same components were used without crude oil to evaluate the consortia in the absence of oil. Perchlorate was added as an oxygen supply for the mixed consortia and the biodegradation processes were stimulated by adsorbing oil on sterilized sand. The experiments were carried out in duplicate and controls of consortia and Zinder medium were included. Samples were processed following a strict protocol and the anaerobic experiments were run in sealed vials and compared to the control experiments. Volatile losses were taken into account by comparing both experiments. The microbial consortia was slightly dependent on the oil storage period, causing oscillations. A reference aerobic bacterial consortium was discussed by Sette et al (2007).

### Hydrocarbon analysis

The hydrocarbon contents from each sampling were subjected to extraction with dichloromethane every 10 (aerobic consortia) or 30 days (anaerobic and mixed consortium). Hydrocarbon fractions were obtained by silica gel chromatography using hexane as eluent for GC-MS analyses. Gas chromatography was carried out with a Hewlett Packard 5890 instrument connected to a Hewlett Packard 5970-MSD mass detector, fitted with a MDN5S coated capilary column (30 m length, 0.25 mm internal diammeter, 0.25 μm film thickness). The GC conditions were: split injection (10:1), with He as carrier gas at 1 mL/min. The data were obtained in both full scan and single ion monitoring (SIM) modes, at 70 eV ionization energy. The temperature program was adapted to each biomarker class. The injector temperature was set at 300°C. The analyses of n-alkanes (*m/z *71) were performed with the temperature program: 80°C (hold 2 min) to 270°C at 4°C/min and at 10°C/min to 300°C (hold 25 min). For the analyses of hopanes and homohopanes (*m/z *191), 25-norhopanes (*m/z *177) and steranes (*m/z *217) the temperature program was: 70°C (2 min) to 190°C at 30°C/min, to 250°C at 1.5°C/min and at 2°C/min to 300°C (hold 20 min). All samples were analyzed in duplicate using 5α-cholestan-3-one, 0.02 μg/mL, as internal standard for the quantitative analyses of biomarker biodegradation. The instrument response was regularly checked by injecting a mixture of n-alkanes.

Biomarker biodegradation ratios were calculated using peak areas from the *m/z *191 and *m/z *217 ion chromatograms. The quantitative analyses were carried out following the methodology previously described by da Cruz et al. (2008).

Gas aliquots were removed every 30 days and the headspace composition was analyzed using gas chromatography-mass spectrometry (GC-MS). The amount of dissolved oxygen (DO) was measured during 60 days each 10 days using a galvanic DO probe from Fisher Scientific (the observed range was 8.2-8.7 mg/L ± 1.5%, temperature 22-24.5°C). The anaerobic assays used resazurin as redox indicator, which remained colorless during the experiment indicating an E' of < -0.11 V. By using the Nernst equation, this redox potential was calculated to correspond to a theoretical oxygen concentration of < 1 pmol/L O_2 _(Zeyer et al. 1986). The oxygen in solution was rapidly measured after opening the flasks at room temperature and atmosphere.

### DNA extraction and 16S rRNA gene libraries

One 16S rRNA gene library was constructed for each of the aerobic (Co_Aer), anaerobic (Co_Ana) and mixed (Co_mix) microbial consortium. Samples for DNA extraction were taken at the end of the biodegradation assay and replicates of each microbial consortium were pooled before molecular biology procedures. DNA extraction of the bacterial consortia was carried out using a protocol based on Groβkopf et al. (1998) and Neria-Gonzáles et al. (2006), with modifications, as follows: the microbial pellets retrieved from enrichments (20 mL-aliquots) were suspended in 600 μL PBS buffer, homogenized by vortex and adding lisozyme to a final concentration of 17 mg/mL. After incubation at 37°C for 1 h, proteinase K and SDS were added (final concentration of 0.7 mg/mL and 2%, respectively) and the solution was incubated at 60°C for 30 min. The tubes were subjected to 3 *freeze-thaw *cycles (2 min in liquid nitrogen followed by 2 min at 65°C). The solution was extracted once with an equal volume of saturated phenol (pH 8.0) and once with an equal volume of chloroform:isoamylic alcohol (24:1). For DNA precipitation, 10% NaCl and 2 volumes of cold ethanol were added to the solution. The *pellet *was washed once with 70% ethanol, dried and suspended in Milli-Q water. For 16S rRNA gene library construction, amplification was performed from total community DNA by using the bacterial primer set 27f and 1100r (Lane 1991). Fifty microliters of the reaction mixtures contained 50 ng of total DNA, 2 U of *Taq *polymerase (GE Healthcare), 0.2 mmol/L^-1 ^of dNTP mix, and 0.4 μmol/L^-^1 of each primer, in 1× *Taq *buffer. PCR amplifications were done using 10 cycles of 1 min at 94°C, 30 s at 60°C, decreasing 0.5°C each cycle, and 3 min at 72°C, followed by additional 10 cycles of 1 min at 94°C, 30 s at 56°C and 3 min at 72°C. Amplicons were pooled from five reactions (~500 ng), purified using *GFX™ PCR-DNA and gel band purification kit *(GE Healthcare) and cloned using the pGEM-T cloning vector kit, according to the manufacturer's instructions (Promega, Madison, WI). All the insert-containing clones were submitted to amplified ribosomal DNA restriction analysis (ARDRA) by digesting M13 amplicons with the enzymes *Hae *III, *Hha *I and *Msp *I, at 37°C for 2 h 30 min. Clones representing distinct ribotypes were selected for DNA sequencing and phylogenetic analysis. The 16S rRNA gene sequences were determined by direct amplification of the inserts from overnight grown-clone cultures with M13 forward and reverse primers and sequencing with the *DYEnamic ET Dye Terminator Cycle Sequencing Kit *for the automated MegaBace 500 system (GE Healthcare), according to the manufacturer's recommendations.

### Sequence analysis

Partial 16S rRNA gene sequences obtained from clones using forward and reverse primers were assembled in a contiguous sequence using the phred/Phrap/CONSED program (Godon et al. 1997; Ewing et al. 1998). Identification was achieved by comparing the contiguous 16S rRNA gene sequences with 16S rRNA sequence data from reference and type strains, as well as environmental clones, available at the public databases Genbank and the RDP (Ribosomal Database Project, Wiscosin, USA) by using BLASTn and RDP sequence match routines. Sequences were aligned using the CLUSTAL X program (Thompson et al. 1997) and analyzed with MEGA software Version 4.0 (Tamura et al. 2007). The evolutionary distances were derived from the sequence-pair dissimilarities, calculated as implemented in MEGA using the DNA substitution model reported by Kimura (1980). The phylogenetic reconstruction was done using the neighbour-joining (NJ) algorithm (Saitou and Nei 1987), with bootstrap values calculated from 1000 replicate runs, using the routines included in MEGA software. The nucleotide sequences determined in this study were deposited at the Genbank database under the accession numbers: FJ493061 to FJ493071.

For diversity analysis of the 16S rRNA libraries, Operational Taxonomic Units (OTU) were defined as distinct ARDRA profiles obtained and total richness as the number of observed OTUs. Coverage values were calculated for each 16S rRNA library using the formula of Good (1953). Rarefaction curves based on ARDRA data were performed using the independent sampling algorithm, implemented in the EcoSim program (Gotelli and Entsminger 2003).

## Results

**Figure 3:**
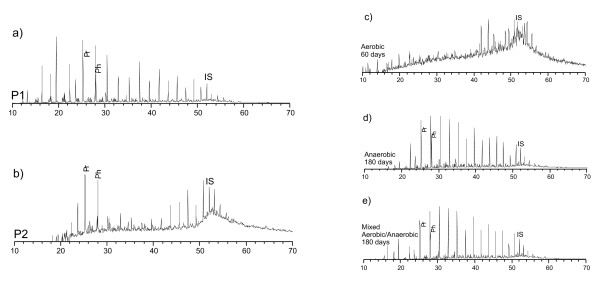
**Total ion chromatograms (GC-MS) of hydrocarbon fractions from oils (Pampo Sul Field, Campos Basin) undergoing *in vitro *(aerobic consortium, 3C; anaerobic consortium, 3D; mixed aerobic and anaerobic consortium, 3E) and *in reservoir *(level 1-2, 3A; level 4, 3B) biodegradation. (Internal Standard (IS), 5α-cholestan-3-one 0,02 μg/μL)**.

Two oils from similar source rocks and from the same basin but displaying different levels of biodegradation were selected for laboratory biodegradation experiments using aerobic, anaerobic and mixed (aerobic/anaerobic) conditions: *i*) a non-biodegraded oil (P1, level 1-2) (Peters and Moldowan 1993), and *ii*) a naturally biodegraded oil (P2, level 4) (Peters and Moldowan 1993). These oils were also used to evaluate *in vitro *and *in reservoir *biodegradation parameters based on specific biomarker ratios (Figure 3, Table 2 and Table 3)

**Table 2:** Biodegradation (%) for *n*-alkanes and isoprenoids by aerobic, anaerobic and mixed consortium.

Hydrocarbon Compound	Biodegradation (%)		
	
	Aerobic (60 days)	Anaerobic (180 days)	Mixed (180 days)
*n*-Tridecane	absent	absent	absent

*n*-Tetradecane	96 ± 0.9	absent	48 ± 1.6

*n*-Pentadecane	> 99 ± 1.60	68 ± 1.5	51 ± 1.4

*n*-Hexadecane	98 ± 2.4	63 ± 1.3	33 ± 1.1

*n*-Heptadecane	> 99 ± 1.1	50 ± 0.9	50 ± 0.8

Pristane	> 99 ± 0.6	45 ± 0.5	46 ± 0.6

*n*-Octadecane	> 99 ± 0.8	54 ± 2.1	42 ± 2.8

Phytane	98 ± 2.7	39 ± 1.2	39 ± 1.8

*n*-Nonadecane	> 99 ± 1.7	55 ± 0.6	57 ± 0.9

*n*-Eicosane	> 99 ± 2.0	45 ± 2.2	42 ± 1.2

*n*-Heneicosane	> 99 ± 1.2	69 ± 1.5	62 ± 1.8

*n*-Docosane	> 99 ± 1.6	49 ± 0.9	47 ± 0.7

*n*-Tricosane	> 99 ± 2.7	60 ± 0.8	60 ± 0.9

*n*-Tetracosane	> 99 ± 1.3	65 ± 1.3	51 ± 1.5

*n*-Pentacosane	> 99 ± 0.9	60 ± 1.8	59 ± 1.6

*n*-Hexacosane	> 99 ± 0.8	64 ± 0.6	44 ± 0.9

*n*-Heptacosane	> 99 ± 2.8	68 ± 1.6	64 ± 1.8

*n*-Octacosane	> 99 ± 2.6	63 ± 1.0	41 ± 2.0

*n*-Nonacosane	> 99 ± 0.6	69 ± 2.0	67 ± 1.0

*n*-Triacontane	> 99 ± 1.5	44 ± 1.1	42 ± 1.3

*n*-Hentriacontane	> 99 ± 1.2	49 ± 1.6	59 ± 1.9

*n*-Dotriacontane	> 99 ± 1.1	50 ± 1.1	42 ± 1.8

**Table 3:** Biomarker ratios for different microbial consortium from laboratory degraded oil and from Pampo Sul field (P2).

Consortia	Biomarkers ratios	Time (days)							
		
		Control	10	20	30	40	50	60	P2
**Aerobic**	**C_28_TT/C_35_H^a^**	0.91	0.93	1.02	1.04	1.23	1.31	1.35	1.10
	**C_29_TT/C_35_H^a^**	1.00	1.30	1.34	1.42	1.62	1.62	1.63	1.47
	**C_35_H/C_30_H^b^**	0.16	0.12	0.12	0.12	0.13	0.14	0.14	0.12
	**Ts/(Ts+Tm)^c^**	0.41	0.32	0.41	0.41	0.41	0.43	0.43	0.32
	**IH^d^**	8.41	8.24	8.13	8.10	7.50	6.90	6.71	7.36
	**Sterane/Hopane^e^**	0.35	0.31	0.38	0.38	0.54	0.79	0.80	0.94

		Control	30	60	90	120	150	180	P2

**Anaerobic**	**C_28_TT/C_35_H**	0.92	1.30	0.91	1.02	0.62	0.60	0.60	1.10
	**C_29_TT/C_35_H**	0.94	1.41	1.22	1.23	0.81	0.73	0.61	1.47
	**C_35_H/C_30_H**	0.15	0.12	0.14	0.14	0.19	0.19	0.18	0.12
	**Ts/(Ts+Tm)**	0.43	0.32	0.43	0.43	0.44	0.44	0.52	0.32
	**IH**	8.31	7.14	7.70	7.94	9.22	9.42	9.63	7.36
	**Sterane/Hopane**	0.73	0.84	0.84	0.81	0.74	0.11	0.10	0.94

		Control	30	60	90	120	150	180	P2

**Mixed Aerobic/Anaerobic**	**C_28_TT/C_35_H**	0.92	1.25	1.52	1.03	1.13	0.84	0.92	1.10
	**C_29_TT/C_35_H**	0.94	1.17	1.30	1.55	1.75	1.43	1.16	1.47
	**C_35_H/C_30_H**	0.15	0.15	0.13	0.12	0.12	0.10	0.14	0.12
	**Ts/(Ts+Tm)**	0.29	0.24	0.27	0.27	0.28	0.28	0.28	0.32
	**IH**	8.31	7.19	6.78	7.47	7.65	7.86	8.57	7.36
	**Sterane/Hopane**	0.36	0.95	1.16	0.72	0.57	0.54	0.07	0.94

**Figure 4:**
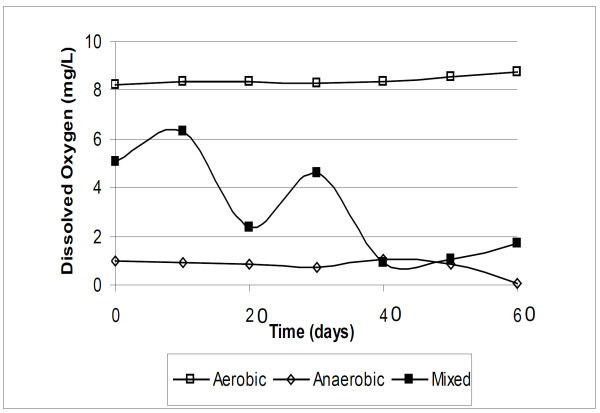
**Dissolved oxygen versus time in laboratory experiments for all consortium**. Dissolved oxygen for aerobic (open square), anaerobic (diamond), mixed aerobic/anaerobic (filled square).

**^a^**Calculated from *m/z *191 mass chromatogram peak areas of the C_28 _- C_29 _tricyclic terpanes (TT) (22*R *+ 22*S*). C_35 _17α.21β(H)-homohopane (C_35_H) (22*R *+ 22*S*) and C_30 _17α.21β(H)-hopane (C_30_H); **^b^**Calculated from *m/z *191 mass chromatogram peak areas of the [C_35 _(22*R*+22*S*)/(C_31_-C_35_)(22*R*+22*S*) homohopanes] × 100; **^c^**Calculated from *m/z *191 mass chromatogram peak areas of the C_27 _17α(H)-22.29.30-trisnorhopane (Tm) and C_27 _18α(H)-22.29.30-trisnorneohopane (Ts); **^d^**Calculated from *m/z *191 mass chromatogram peak areas of the [C_35 _(22*R *+ 22*S*)/(C_31_-C_35_) (22*R *+ 22*S*) homohopanes] × 100; **^e^**Calculated from *m/z *217 mass chromatogram peak areas of the C_27_, C_28 _and C_29 _ααα (20*R *+ 22*S*) and αββ (20*R *+ 22*S*) steranes and from *m/z *191 of the C_29_-C_33 _17α(H)-hopanes (20*R *+ 22*S*);

Oxygen content was measured every 10 days for 60 days, showing low oxygen content in anaerobic experiments (8-9 mg/L) and oscillating content in experiments with mixed consortia (between ~0.8 mg.L^-1 ^and ~6 mg.L^-1^) (Figure 4).

**Figure 5:**
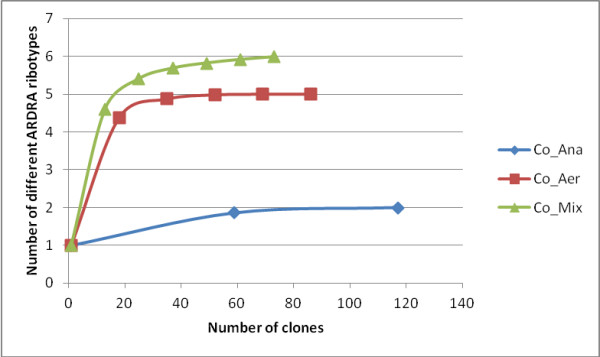
**Rarefaction curves of observed OTU richness (number of different ARDRA ribotypes) in bacterial 16S rRNA libraries from consortia samples**.

Consortia of microorganisms present in aerobic, anaerobic and mixed consortium biodegradation experiments were assessed by rRNA 16S. Community analysis of the microbial consortia showed that bacterial populations were affiliated to the genera *Achromobacter *(43%), *Bacillus *(32%), *Brevibacterium *(10%) and *Mesorhizobium *(9%) in the aerobic consortium, *Bacillus *(97%) and *Acinetobacter *(3%) in the anaerobic consortium, and *Stenotrophomonas *(49%), *Bacillus *(16%), *Rhizobium *(11%), *Achromobacter *(10%) and *Brevibacterium *(10%) in the mixed consortium (Table 4). High coverage values were found, demonstrating that the bacterial diversity of the consortia was almost fully covered by the clone libraries (Table 4). Rarefaction curves were consistent with the coverage values found and reached saturation for all 16S rRNA libraries, specially for Co_Aer and Co_Ana libraries, indicating that the sampling effort was sufficient to reveal all bacterial species present in the consortia under study (Figure 5).

**Table 4:** Coverage values and composition of bacterial 16S rRNA gene clone libraries from the aerobic (A), anaerobic (B) and mixed (C) consortium.

Microorganism	A	B	C
Bacillus sp.	18%	89%	14%
Brevibacterium sp.	18%		14%
Mesorhizobium	5%		
Achromobacter sp.	58%		11%
Acinetobacter		11%	
Stenotrophomonas sp.			50%
Rhizobium sp.			6%
Uncultured bacterium			5%

## Discussion

In our assays the aerobic consortium selectively degraded *n*-alkanes and isoprenoids (biodegradation rates of 36 to 99% in 60 days), with decreasing ratios of pristane (Pr)/phytane (Ph), increasing ratios of Pr/*n*C_17 _and Ph/*n*C_18_, and an even-to-odd predominance over all the biodegradation process. The anaerobic (micro) consortium showed a slower depletion rate of *n*-alkanes and isoprenoids (39 to 69% in 180 days) and an odd-to-even predominance. The mixed aerobic/anaerobic consortia alternated *n*-alkane biodegradation preferences (33 to 64% in 180 days) and the even-to-odd or odd-to-even preferences with lower biodegradation rates than with the pure aerobic consortium (Table 2). It is commonly accepted that odd number n-alkane preference is associated with low oil thermal maturity; however these experimental data reveal that the alkane carbon number predominance can change with the type of biodegradation and should be interpreted with caution.

Headspace monitoring for 270 days (every 10 days) of the anaerobic and mixed consortia detected acetic acid (Heuer et al. 2010, 120 days of biodegradation) and CO_2 _(270 days of biodegradation) but no methane, indicating the presence of acetogenic bacteria and the absence of methanogenic archaea in the microbiota Zengler et al. (1999) showed that the process of CH_4 _formation is very slow, taking about 150 days to obtain detectable amounts of CH_4_.

Biodegradation with the aerobic consortia increased the ratios of tricyclic terpane (TT) [C_28 _(TT)/C_35 _17α,21β-homohopane (C_35_H), C_29 _tricyclic terpane (TT)/C_35 _17α,21β-homohopane (C_35_H)] with increasing biodegradation time, revealing a preferencial depletion of (*R+S*) C_35 _hopane (> 82% depletion) in relation to tricyclic terpane (22 and 17% depletion of C_28 _and C_29 _TT, respectively) (Table 3). These assays confirmed the selective biodegradation of 22*R *homohopane isomers. The anaerobic experiment revealed that these ratios decreased, suggesting a preferential depletion of tricyclic terpane (40 and 38% depletion for C_28 _and C_29 _TT, respectively) relative to C_35 _hopane (36% depletion). The aerobic consortia degraded 0 to 22% C_23_-C_31 _TT, whereas the anaerobic consortia degraded 19 to 40% of such compounds. This effect reveals that oils with lower tricyclic/17α-hopane ratios, usually interpreted as more mature oils, could, in fact, be the consequence of an anaerobic microbiota biodegradation operating at higher temperatures.

The homohopane index decreased for aerobic consortium, showing that C_35 _is more biodegradable than C_31_-C_34 _homohopanes, with preferential biodegradation of the higher molar-mass 22*R *epimers C_35 _> C_34 _> C_33 _> C_32 _> C_31 _extended hopanes. Our data are similar to the results of the laboratory culture experiments made by Goodwin et al. (1983) but opposite to the laboratory studies conducted by Bost et al. (2001). These apparent conflicting results indicate that experiments with different consortia produce different biomarker parameters. Therefore the coherence in biodegradation parameters applied to petroleum analyses for the last 60 years indicate that the microbiota in most reservoirs are consistent. The homohopane index increased during the anaerobic biodegradation and indicates a preferential biodegradation of the lower molar mass homohopane 22*R *C_31 _> C_32 _> C_33 _> C_34 _> C_35_, suggesting that C_35 _is more resistant to the biodegradation process. The bioresistance of higher-hopane homologs has been reported in different oils (Moldowan and McCaffrey 1995; Peters and Moldowan 1991).

The experiment with mixed consortia, revealed an oscillating content of oxygen with periods of microaerobicity, at ~0.8 mg/L and aerobicity at ~6 mg/L of oxygen. These experiments were performed in closed vessels, therefore the oxygen was generated and consumed by the microbiota in alternated lifecycles (Figure 4). The biodegradation preference for all biomarkers was unusual, with aerobic characteristics (increasing C_28_TT from 30 to 90 days, Table 3) alternating with anaerobic ones (decreasing C_28_TT from 90 to 120 days, Table 3). The homohopane index decreased in the first 60 days and increased from then on until the assay was completed (180 days), with an unusual homohopane biodegradation preference C_33 _> C_32 _> C_31 _> C_34 _> C_35_Munoz et al. (1997) showed 25% depletion of C_35 _pentakishomohopane and only 3% depletion of C_31 _homohopanes in soils exposed to an oil spill for eight years. Thus the controversial relative suceptibility of homohopanes to biodegradation, observed in the field and in laboratory experiments (Goodwin et al. 1983; Bost et al. 2001; Moldowan and McCaffrey 1995; Peters and Moldowan 1991; Munoz et al. 1997) might be assigned to different microbiota, aerobic, anerobic or both, when dealing with oils of equal origin and reservoir matrix. Consequently our experiments validate the C_35 _homohopane index traditionally used to evaluate the oxic or suboxic environment, linking this index to aerobic, anaerobic and mixed biodegradations (Obermajer et al. 2000).

The norhopanes 18α (H)-22,29,30-trisnorneohopane (Ts) and 17α(H)-22,29,30-trisnorhopane (Tm) remained almost unaltered throughout the experiment, with a small Ts/Tm ratio increase, indicating that Tm is more susceptible to biodegradation in all processes (aerobic, anaerobic and mixed).

During aerobic experiments the steranes (67% depletion) were less degraded than hopanes (87% depletion) and an increase of the sterane/hopane ratio was observed. Under anaerobic conditions sterane biodegradation (80% depetion) occured before that of hopane (67% depletion), decreasing the sterane/hopane ratio (Table 3). Mixed and aerobic consortium preferentially biodegraded steranes relative to hopanes (30 to 90 days) however this relative susceptibility was inverted over the next 60 days (120 to 180 days), which is consistent with anaerobic biodegradation. Sterane preferencial biodegradation was equal under both aerobic and anaerobic consortium, ααα 20*R >*> αββ 20*R *+ αββ 20S ≥ ααα 20*S *> > diasteranes, and opposite for mixed consortia, αββ 20*R *+ αββ 20S > ααα 20*R *> ααα 20*S *> > diasteranes with increasing carbon number C_27 _> C_28 _> C_29 _for all experiments. The relative susceptibility of these isomers varies considerably and depends on the microbiota involved, as shown by the present results. Therefore the 20S/(20S + 20R) ratio used as a thermal maturation parameter should be used with caution.

**Figure 6:**
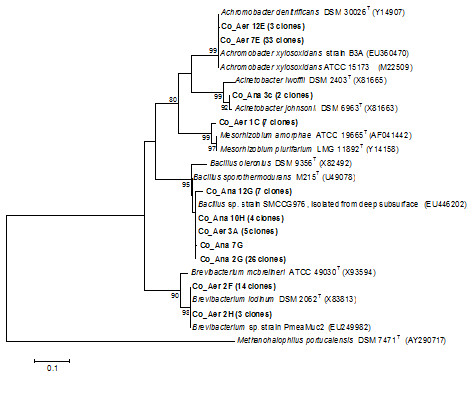
**Phylogenetic analysis of partial 16S rRNA gene sequences of clones from aerobic (Co_Aer) and anaerobic (Co-Ana) consortium and related species using the Kimura 2p evolutionary model and the *neighbor joining *method for tree reconstruction**. Bootstrap values (1000 replicate runs, shown as %) greater than 70% are listed. Numbers in brackets correspond to additional clones showing ≥ 97% similarity with the clone represented in the branch. GenBank accession numbers are listed after species names. *Methanohalophilus portucalensis *was used as the outgroup.

To link biodegradation to specific biodegrading microbes the 16S rRNA gene sequences of the all three microbiota were analyzed indicating the most abundant genera in each microbiota and their phylogenetic tree (Table 4, Figure 6). The bacterial genera detected have all been previously described in the literature as hydrocarbon degraders and/or being associated with oil field environments (Bieszkiewicz et al. 1998; Orphan et al. 2000; Chaillan et al. 2004; Pineda-Flores et al. 2004; Nestler et al. 2007). The *Achromobacter *strains that dominate the aerobic consortium were phylogenetically related to the species *A. xylosoxidans *and *A. denitrificans *(Figure 6). Phylogenetic analysis revealed that *Bacillus*-related clones from both aerobic and anaerobic consortium were closely related to *Bacillus sporothermodurans *(Figure 6), which is able to produce highly heat-resistant endospores and has already been found in oil fields (Petterson et al. 1996). Our results corroborate previous literature data on the isolation and biodegradation ability of *Bacillus *spp. from Brazilian reservoirs (Sette et al. 2007; Sebastián 1999). The predominance of *Bacillus *spp. in the anaerobic consortium could be explained by the fact that some species belonging to this genus are facultative anaerobes (Cunha et al. 2006), capable of shifting their respiration mechanism from aerobic to anaerobic conditions. These bacteria could be responsible for hydrocarbon biodegradation under anaerobic conditions by using sulfate or other salts as electron acceptors, via a dissimilatory sulfate reduction pathway (Clements et al. 2002).

These controlled *in vitro *experiments (anaerobic, aerobic and both aerobic/microaerobicity conditions) provide a unique ensemble of data that were used in an attempt to explain controversial observations of *in reservoir *biodegradation and that attested to some biodegradation parameters. The evidence suggests that aerobic biodegradation is mainly responsible for the depletion of the linear hydrocarbons with an even-to-odd carbon preference, although switching to an anaerobic biodegradation, with odd-to-even carbon preference, was observed. The mixed (aerobic and anaerobic consortium) displayed periods of C-even preference and periods of C-odd preference, indicating the existence of alternating aerobic and anaerobic lifecycles. A comparative biodegradation of terpanes, hopanes and steranes under aerobic and anaerobic conditions, Table 3, indicate that some of the controversial biomarker parameters can be easily explained by taking into consideration the microbiota and oxygen content. These results represent an important step in understanding reservoir biodegradation, revealing that anaerobic and aerobic biodegradation can indeed occur without external input of oxygen.

## Competing interests

The authors declare that they have no competing interests.

## Authors' contributions

AJM research team of graduate students GFC, and CFFA carried out the biodegradation experiments, microorganism isolation, hydrocarbon analyses and drafted the manuscript. VMO reasearch team of graduate and post graduate students BMD, INSG and SPV carried out the DNA extraction and 16S rRNA gene libraries. EVSN provided the petroleum samples. EVSN, AJM and VMO are responsible for supervision and rationale of all experiments. All authors read and approved the final manuscript.
